# Aquila: Efficient In-Kernel System Call Telemetry for Cloud-Native Environments

**DOI:** 10.3390/s25216511

**Published:** 2025-10-22

**Authors:** Juyong Shin, Jisu Kim, Jaehyun Nam

**Affiliations:** 1 Department of AI-Based Convergence, Dankook University, 152, Jukjeon-ro, Suji-gu, Yongin-si 16890, Republic of Korea; juyongshin@dankook.ac.kr (J.S.); imjs0807@dankook.ac.kr (J.K.); 2 Department of Computer Engineering, Dankook University, 152, Jukjeon-ro, Suji-gu, Yongin-si 16890, Republic of Korea

**Keywords:** cloud-native security, system call telemetry, eBPF monitoring

## Abstract

System call telemetry is essential for understanding runtime behavior in cloud-native infrastructures, but existing eBPF-based monitors suffer from high per-event overhead, unreliable delivery under load, and limited context for correlating multi-step activities. These issues reduce scalability, create blind spots in telemetry streams, and complicate the analysis of complex workload behaviors. This work presents Aquila, a lightweight telemetry framework that emphasizes efficiency, reliability, and semantic fidelity. Aquila employs a dual-path kernel pipeline that separates fixed-size metadata from variable-length attributes, reducing serialization costs and enabling high-throughput event processing. It introduces priority-aware buffering and explicit drop detection to retain loss-sensitive events while providing visibility into overload conditions. In the user space, kernel traces are enriched with Kubernetes metadata, mapping low-level system calls to pods, containers, and namespaces. Evaluation under representative workloads shows that Aquila improves scalability, reduces event loss, and enhances the semantic completeness of system call telemetry compared with existing approaches.

## 1. Introduction

System call monitoring provides fine-grained visibility into interactions between user processes and the operating system kernel, forming a foundational capability for understanding runtime behavior and diagnosing performance issues in modern computing environments. By exposing low-level execution traces, system call telemetry enables accurate characterization of workload dynamics and post-hoc analysis of runtime behaviors. In cloud-native infrastructures, where workloads are decomposed into microservices and orchestrated dynamically across multi-tenant clusters, this visibility becomes particularly important [1]. Cloud-native systems exhibit rapid scaling, frequent container churn, and highly heterogeneous runtime environments, creating a need for monitoring solutions that can handle high event volumes efficiently while preserving semantic context across workloads [2]. As a result, system call telemetry has become a foundational input to observability pipelines in modern containerized infrastructures.

Traditional approaches to system call monitoring relied on either user-space interception or kernel modules, both of which introduced significant operational and performance constraints. User-space interception mechanisms (e.g., ptrace [3]) introduced prohibitive context-switching costs because every system call triggered additional user–kernel transitions, making these methods unsuitable for high-throughput environments. Kernel-integrated logging facilities such as auditd [4] reduced interception overhead but provided only coarse-grained filtering, forcing operators to trade precision for scalability when processing large volumes of events. Kernel instrumentation frameworks such as SystemTap [5] and LTTng [6] offered finer-grained visibility but required custom modules or debug symbols, introducing portability and stability concerns that limited their adoption in production environments. These limitations created a gap between the demand for high-fidelity telemetry and the capabilities of legacy monitoring solutions.

The emergence of the extended Berkeley Packet Filter (eBPF) [7] fundamentally changed the design space for system call monitoring by enabling safe, programmable execution paths inside the Linux kernel. eBPF programs are statically verified for safety, dynamically attachable to system call tracepoints or Linux Security Module (LSM) hooks, and capable of in-kernel filtering and enrichment prior to exporting events [8]. Building on these primitives, general-purpose tools such as bpftrace [9] provide flexible tracing capabilities for performance analysis and debugging, while container-aware systems, including Tracee [10], Falco [11], and Tetragon [12], extend telemetry collection with workload context, rule-based filtering, and integration with orchestration platforms. These systems illustrate the progression from basic system call tracing toward observability pipelines capable of correlating kernel-level events with containerized workloads.

Despite these advances, existing eBPF-based telemetry frameworks face persistent challenges in large-scale cloud-native deployments. First, they impose substantial per-event processing overhead because every system call must be serialized inside the kernel, transferred through shared buffers, and reconstructed in user space. This overhead accumulates rapidly under high-frequency workloads such as CI/CD pipelines or I/O-heavy microservices [13]. Second, event delivery remains unreliable because most frameworks rely on bounded buffers (e.g., perf or ring buffers) that silently discard events when production rates exceed consumer throughput [14]. Such data loss reduces the completeness of telemetry streams and makes it difficult to reason about system behavior under overload conditions. Third, current solutions provide only a limited semantic context for multi-step runtime activities [15]. They typically capture system calls as isolated records rather than correlated sequences, making it challenging to reconstruct the higher-level workflows or runtime interactions that emerge across processes, containers, and namespaces.

This paper presents **Aquila**, an in-kernel system call telemetry framework designed to deliver efficient, reliable, and semantically comprehensive rich observability for cloud-native workloads. Aquila employs a dual-path kernel pipeline that separates fixed-size metadata from variable-length attributes, reducing serialization overhead while preserving complete event fidelity. It incorporates priority-aware buffering and explicit drop detection to maintain visibility into data loss while guaranteeing the retention of high-value telemetry under overload conditions. Furthermore, Aquila enriches kernel-level traces with container and Kubernetes metadata in the user space, aligning low-level system call events with pods, containers, and namespaces to integrate seamlessly with existing observability toolchains. Through these mechanisms, Aquila provides scalable and context-rich system call telemetry without introducing application-level instrumentation or kernel modifications.

In practice, Aquila benefits both developers and system administrators. Developers can leverage correlated system call workflows to debug multi-step execution paths, such as process creation chains or configuration file accesses, without intrusive instrumentation. System administrators gain the ability to attribute security-sensitive system calls directly to pods and namespaces, accelerating incident response and root-cause analysis. In contrast, existing tools often expose raw system call streams with higher event loss, leaving practitioners to manually reconstruct context after the fact.

**Contributions.** This paper makes the following key contributions:

- It analyzes the limitations of existing eBPF-based telemetry frameworks in terms of event processing overhead, reliability under load, and lack of semantic correlation for multi-step runtime activities.
- It introduces a dual-path event pipeline that separates fixed-size metadata from variable-length attributes, reducing per-event processing costs and enabling high-throughput reconstruction in user space.
- It incorporates priority-aware buffering and explicit drop detection using per-CPU counters and sequence numbers to maintain loss visibility and preserve essential telemetry records under overload conditions.
- It implements and evaluates a prototype of Aquila under realistic workloads, demonstrating improved scalability, reliability, and semantic expressiveness compared with existing approaches.

**Paper Outline.** Section 2 reviews system call telemetry in cloud-native environments. Section 3 and Section 4 describe the architecture and implementation of Aquila. Section 5 presents experimental results, Section 6 discusses related work, and Section 7 concludes the paper.

## 2. Background and Motivation

System call monitoring offers fine-grained visibility into program execution and remains central to security, scalability, and observability in cloud-native infrastructures. Traditional techniques, such as user-space interception or kernel modules, enabled system call tracing but incurred substantial performance overhead, limited maintainability, and operational complexity. The introduction of eBPF alleviated many of these limitations through safe in-kernel programmability, yet large-scale deployments continue to face challenges involving performance, reliability, portability, and semantic expressiveness.

### 2.1. System Call Monitoring in Cloud-Native Environments

Cloud-native architectures rely on microservices decomposition, container orchestration, and elastic scaling across multi-tenant infrastructures. In Kubernetes clusters, thousands of short-lived containers may be instantiated on shared worker nodes, each issuing system calls for process creation, file access, network communication, and privilege management. These system calls represent the most detailed runtime signals available for characterizing workload behavior. Observability systems use system call telemetry to construct execution timelines [16] and to analyze workload interactions at high resolution [17].

Meeting these objectives imposes stringent requirements. Monitoring infrastructures must sustain millions of events per second while introducing minimal overhead, preserve semantic accuracy and reliability despite multi-tenancy and container abstraction layers, and remain compatible with orchestration platforms where workloads frequently migrate across nodes. Realistic workloads such as CI/CD pipelines, container image construction, and I/O-intensive microservices exhibit highly variable system call rates that often generate transient bursts, revealing the scalability limitations of existing monitoring frameworks and highlighting the need for more efficient designs.

### 2.2. Limitations of Legacy System Call Monitoring Approaches

Before in-kernel programmability became widely available, system call monitoring primarily employed two approaches: user-space interception and kernel-level instrumentation. User-space interception, most prominently via ptrace [3], captured system calls at the user–kernel boundary. Although easy to deploy, this method imposed prohibitive overhead and complexity because each system call triggered additional context switches, making it unsuitable for high-throughput and scalability-critical environments [18]. Kernel logging via auditd [4] provided an alternative by integrating monitoring directly into the kernel, but its coarse-grained filtering often forced administrators to choose between overwhelming log volumes and incomplete visibility [19].

Kernel-level instrumentation frameworks such as SystemTap [5] and LTTng [6] sought to reduce the costs of user-space interception by inserting dynamic probes into kernel code paths. These systems enabled moderately granular tracing but required custom modules or debug symbols, introducing operational complexity and limiting adoption in production environments where kernel stability is critical. Commercial and open-source tools such as the early version of sysdig [20] adopted a similar strategy by deploying kernel modules to extract detailed system call traces with configurable filters, but this approach expanded the kernel’s attack surface and introduced significant maintenance and portability concerns.

These legacy approaches, as summarized in Table 1, embodied persistent trade-offs. Mechanisms that were simple to deploy, such as ptrace, suffered from severe performance penalties, while solutions that reduced runtime overhead, such as SystemTap or sysdig, imposed kernel dependencies and operational risks. Consequently, none of these techniques satisfied the requirements of modern cloud-native environments, which demand high-fidelity telemetry, low overhead, strong isolation, and operational simplicity.

### 2.3. Emergence of eBPF-Based Monitoring Frameworks

The introduction of eBPF [7] fundamentally transformed the design of system call monitoring by enabling safe and programmable execution paths inside the kernel. Unlike earlier mechanisms relying on kernel modules or user-space interception, eBPF programs are verified for memory safety and guaranteed termination before deployment [21]. This model allows programs to attach dynamically to system call entry and exit points or tracepoints and execute custom logic inline with kernel events. By reducing reliance on external modules, eBPF minimizes runtime overhead, preserves kernel stability, enhances portability, and allows monitoring logic to be adapted with greater flexibility and without disruptive kernel modifications. In-kernel filtering and enrichment further ensure that only relevant events are exported to the user space, improving scalability and efficiency.

Building on these primitives, several frameworks have emerged to support observability in cloud-native environments. General-purpose tools such as bpftrace [9] provide high-level interfaces enabling developers and operators to prototype and attach probes for debugging, profiling, and performance analysis. Security-oriented frameworks incorporate richer contextual information for domain-specific features. For example, Tracee [10] augments telemetry with container and Kubernetes metadata to support workload attribution across multi-tenant environments. Falco [11] integrates rule-based filtering for continuous evaluation of system call streams against defined security rules to generate alerts on anomalous runtime behavior. More recent systems, such as Tetragon [12], enrich observability by associating kernel events with namespaces and container identities at collection time, offering more accurate attribution of low-level kernel activity to higher-level workload identities.

These developments illustrate the evolution from basic system call tracing toward context-aware observability integrated with cloud-native abstractions. Early frameworks emphasized post-hoc visibility by streaming raw events to user space, producing valuable audit data but limited interpretability. Later systems introduced in-kernel filtering and metadata enrichment to reduce overhead and improve precision, aligning outputs more closely with orchestration platforms. However, this evolution also introduced trade-offs: enrichment and filtering increase per-event cost, buffering mechanisms remain prone to overload, and correlating multi-step runtime behaviors often requires user-space pipelines. These limitations motivate the need for improved designs that deliver scalability and semantic fidelity in dynamic environments.

### 2.4. Key Challenges in Current eBPF-Based Solutions

Despite significant improvements over legacy methods, current eBPF-based telemetry frameworks face several persistent challenges in large-scale cloud-native clusters. These limitations manifest as both technical inefficiencies and reliability risks.

The first challenge is performance overhead. eBPF avoids the high context-switching costs of user-space interception, but each system call event still requires processing in both kernel and user space. This involves constructing event records in the kernel, transferring them into shared buffers, and subsequently deserializing and enriching them in user space. Under high-frequency workloads generating millions of system calls per second, such as CI pipelines, image builds, or I/O-intensive services, these operations saturate CPU budgets and delay critical workloads [22]. Even lightweight tasks such as string handling for file paths, assembling container identifiers, and serializing payloads accumulate into measurable contention that limits scalability and degrades overall cluster performance.

The second challenge is event loss under overload. Current frameworks depend on bounded buffers, such as perf or ring buffers, to transfer events to user space. When production rates exceed consumption capacity, entries are overwritten or dropped without prioritization [14]. This creates critical blind spots in audit trails and undermines the completeness of workload analysis. It also introduces a significant security concern: adversaries can deliberately flood the system with benign calls to mask rare but critical events, such as a suspicious execve() invocation. This silent nature of such drops leaves system operators unaware until after data gaps have already affected downstream pipelines [23].

The third challenge is limited semantic context for complex behaviors. Most frameworks capture system calls only after completion, which suffices for raw observability but fails to represent relationships across sequences of calls. Many runtime behaviors emerge across multiple processes or namespaces and cannot be understood by isolated events alone [24]. However, eBPF programs have limited capabilities for maintaining long-lived state or correlating events across time and processes. As a result, multi-step interactions are typically reconstructed in user-space pipelines, where analysis incurs higher latency and may suffer from incomplete consistency.

As summarized in Table 2 and Table 3, eBPF-based monitoring frameworks have advanced well beyond legacy solutions but remain constrained by three systemic limitations: high per-event overhead reducing scalability, unreliable delivery compromising completeness, and insufficient semantic context limiting analysis of complex behaviors. Overcoming these challenges requires architectural designs that balance kernel-space efficiency with user-space flexibility, ensuring both performance and fidelity in modern cloud-native deployments.

## 3. Aquila Design

The design of Aquila aims to overcome the systemic limitations identified in current eBPF-based monitoring systems. It introduces an architecture that balances in-kernel efficiency with user-space flexibility, while ensuring reliable delivery and context-rich telemetry. This section describes the guiding requirements, the detailed architecture, and operational workflows that collectively enable the system to deliver scalable, reliable, and semantically rich monitoring in cloud-native environments.

### 3.1. Design Requirements

The practical constraints observed in current eBPF-based systems motivate four key requirements for robust deployment:

R1: Low overhead and scalability. The system must minimize per-event processing costs and sustain workloads generating millions of system calls per second. Efficient partitioning of enrichment and serialization tasks between kernel and user space is essential to prevent CPU contention and preserve scalability under latency-sensitive workloads.

R2: Reliable event delivery. The system must ensure loss-aware and backpressure-resilient buffering to prevent silent data loss that could undermine behavioral analysis accuracy. Security-critical events must be preserved with strict priority even under bursty or adversarial workloads that deliberately attempt to saturate the monitoring pipeline.

R3: Semantic context and correlation. The system must capture causal relationships across processes and system calls by maintaining a lightweight in-kernel context and temporal linkage, allowing multi-step behaviors to be reconstructed accurately rather than treating events as isolated, post-execution traces.

R4: Cloud-native integration. Collected events must be mapped to higher-level abstractions such as pods, containers, and namespaces. The system should avoid invasive kernel dependencies and integrate seamlessly with Kubernetes primitives to enable automated deployment and updates.

### 3.2. Overview

Aquila employs a modular architecture designed to meet the four requirements outlined above. It comprises three layers: an in-kernel event pipeline for efficient data capture, a reliability layer for prioritized buffering and explicit loss detection, and an enrichment layer that aligns kernel-level telemetry with cloud-native abstractions. This separation of concerns ensures that scalability, reliability, and contextual accuracy are addressed independently yet operate cohesively as a single pipeline.

**Figure 1:**
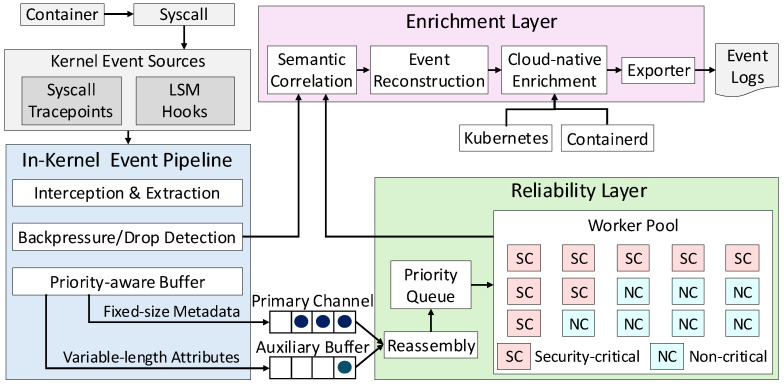
Overall architecture of Aquila with in-kernel event capture, priority-aware buffering, loss detection, and user-space enrichment for Kubernetes-aware telemetry.

Figure 1 illustrates the overall architecture of Aquila. The event pipeline consists of three complementary components. The interception and classification module attaches probes to both system call tracepoints and LSM hooks, providing visibility into post-execution traces as well as early kernel activities. This dual attachment ensures that both completed operations and their initiation points are observed, improving temporal accuracy for workload analysis.

The transmission module separates fixed-size metadata (e.g., Linux namespace IDs, PID/TID, system call ID, parameters) into a high-throughput ring buffer, while variable-length attributes such as file paths and arguments are placed in an auxiliary buffer. Both streams are linked via a 64-bit event identifier, allowing the collector to reassemble complete events. This design sustains high throughput on the hot path while preserving full event fidelity.

Finally, the reliability and enrichment module applies priority-based buffering and explicit loss reporting within the kernel, while user-space enrichment resolves container and Kubernetes metadata for each event. This design preserves high-priority events under overload, exposes loss statistics for accurate monitoring, and expresses kernel-level telemetry in terms meaningful to cluster operators, ensuring efficient capture, robust delivery, and seamless integration with existing observability stacks.

The workflow can be illustrated using the execve system call. When invoked, both the system call tracepoint and the security_bprm_check LSM hook are triggered. At this point, fixed-size metadata, including system call identifiers, process IDs, and Linux namespace information, is batched into the ring buffer, while variable-length attributes such as binary paths and arguments are collected in parallel and stored in the auxiliary channel, linked via event identifiers. Events classified as high priority are directed to a reserved buffer to guarantee delivery even under overload, ensuring that essential execution information remains available. Low-priority events may be discarded if necessary to maintain throughput and prevent system-wide slowdowns. Whenever drops occur, per-CPU counters and embedded sequence numbers allow the user-space collector to quantify the exact scope and timing of data loss, attributing it to overload rather than silent failures. After transmission, the collector reconstructs complete events by joining metadata with associated attributes and enriches them with container runtime information to map processes to pods, containers, and namespaces. This enriched view bridges low-level kernel activity with high-level workload abstractions, allowing operators to correlate runtime behavior with orchestration-level entities.

Overall, this workflow demonstrates how in-kernel capture, priority-aware buffering, explicit loss detection, and user-space enrichment combine to provide high-throughput, context-rich monitoring that delivers greater reliability, scalability, and observability while remaining robust under dynamic and large-scale cloud-native workloads.

### 3.3. Kernel-Space Event Processing

The kernel-space pipeline in Aquila implements a multi-stage mechanism that separates metadata capture, attribute extraction, buffering, and loss accounting into coordinated steps. This modular design bounds the overhead of system call monitoring while guaranteeing accurate reconstruction and explicit awareness of data loss.

**Figure 2:**
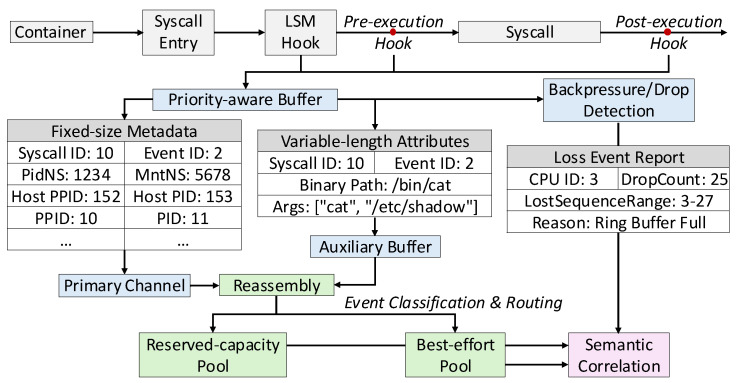
In-kernel pipeline of Aquila. System call and LSM hook events pass through priority-aware buffering, with fixed-size metadata and variable-length attributes handled via separate channels. Events are classified into reserved-capacity or best-effort pools, while backpressure detection and loss reporting enable reliable and context-rich telemetry under high load.

As shown in Figure 2, processing begins at the interception point. When a system call or LSM hook is triggered, kernel probes extract fixed-size metadata such as the system call identifier, process ID, thread ID, and namespace information. These values are placed into the global ring buffer, which serves as the primary channel for fast-path transmission. To avoid synchronization bottlenecks across CPUs, each record embeds a sequence counter that is incremented on a per-CPU basis, ensuring uniqueness and reliability without requiring global locks. Cache-line alignment keeps updates localized to the originating CPU’s cache, while batched commits group multiple records into a single flush, improving efficiency and amortizing the cost of user–kernel transitions and further reducing overhead.

Variable-length attributes are handled separately. For system calls such as execve, the absolute binary path and command-line arguments are retrieved from kernel memory using BPF helper functions. Rather than forcing this data into the fixed-size ring buffer, the pipeline stores it in a dedicated auxiliary buffer. To preserve consistency, each metadata record carries a 64-bit event identifier constructed from a per-CPU counter concatenated with process-specific information. Entries in the auxiliary buffer are tagged with the same identifier, allowing user space to reassemble complete events without requiring global synchronization or ordering locks.

Priority-aware buffering. After metadata capture, the pipeline applies priority separation at the buffer level. Two logical buffer pools are maintained: a reserved-capacity pool for security-critical system calls and a best-effort pool for background events. Classification occurs at capture time using a system call identifier lookup table embedded in the BPF program. Security-critical system calls (Table 4) such as clone(3), execve(at), and open(at) are routed into the reserved pool, ensuring reliable delivery even under heavy load. Non-critical system calls, such as read or write, are directed to the best-effort pool, where drops may occur if saturation is reached. For example, during I/O-intensive workloads, repetitive read() calls may be discarded to preserve essential events such as execve() or openat(). This selective policy guarantees that security-critical activities are retained, while routine bulk operations may be safely sacrificed to maintain throughput.

Backpressure and drop detection. To avoid silent monitoring blind spots, the kernel explicitly accounts for loss events. If buffer allocation fails, the pipeline increments a per-CPU drop counter stored in a BPF map. These counters are updated atomically without locks, since each CPU modifies only its local entry. In addition, every metadata record embeds a monotonically increasing sequence number. When user space receives records, it can detect continuity gaps; for example, a transition from sequence 100 to 103 indicates that entries 101 and 102 were lost. Counters provide aggregate loss statistics, while sequence numbers reveal where specific events were dropped. The combination of these mechanisms prevents silent failures, improves efficiency, and enables operators to quantify monitoring reliability and scalability with precision.

By combining per-CPU batching for metadata, auxiliary channels for attributes, system call-aware prioritization, and dual-mode drop detection, Aquila ensures that kernel-space event processing remains efficient, resilient, and transparent under diverse workloads.

### 3.4. Semantic Correlation and Pre-Execution Visibility

**Figure 3:**
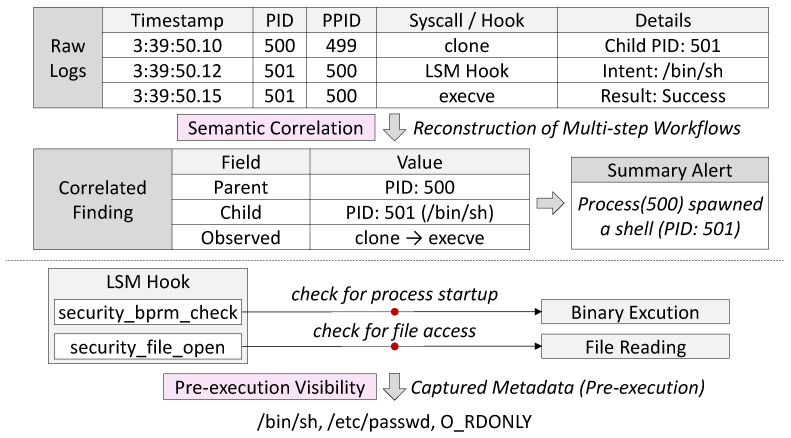
Semantic correlation and pre-execution visibility in Aquila. Multi-step workflows such as clone → execve are reconstructed from raw system call and LSM hook events. Pre-execution hooks (e.g., security_bprm_check, security_file_open) capture process startup and file access intent, enabling early attribution and context-rich alerts before execution completes.

Raw event capture alone cannot expose the higher-order relationships that reveal complex behaviors. As illustrated in Figure 3, Aquila extends observability by incorporating mechanisms that correlate system calls into multi-step workflows and by leveraging pre-execution LSM hooks that expose kernel activity before it is finalized.

Correlation of multi-step behaviors. Many workload characteristics emerge not from individual system calls but from their ordered combination. For example, process creation, namespace switching, and subsequent file operations together describe a unit of work that is more informative than any single event. Aquila leverages the process and thread identifiers (PIDs and TIDs) as well as sequence numbers assigned to each system call record. By linking events that share the same identifiers and reconstructing causal relationships across related system calls in real time. For instance, a sequence such as clone() creating a child process, followed by setns() to join a container namespace, then execve() to launch a new binary, and finally openat() to access a configuration file is correlated as a single workflow. Rather than viewing these calls as isolated events, Aquila reconstructs them into a coherent execution chain, enabling operators to recognize higher-level runtime behaviors such as container initialization or service startup. By keeping per-event metadata fixed-size and performing correlation in user space, the kernel-side logic remains simple and bounded, ensuring predictable memory usage and enabling efficient reconstruction of higher-level execution contexts and workload patterns.

Pre-execution hooks. By attaching eBPF programs to LSM hooks, Aquila observes critical kernel operations before they complete, capturing runtime intent in addition to post-execution effects. For example, security_bprm_check is invoked prior to binary execution, allowing the system to record the command path, arguments, and associated process context before the process is spawned. Similarly, security_file_open provides visibility into file access requests before descriptors are allocated, enabling early attribution of file interactions to specific processes and containers. As these hooks operate in a non-blocking, low-overhead manner, they preserve system performance while enriching telemetry with pre-execution semantics. This capability extends observability beyond completed actions, ensuring that monitoring pipelines can reconstruct both the intent and outcome of sensitive runtime operations with high fidelity.

These semantic correlation and pre-execution hooks allow Aquila to link system calls into meaningful workflows while capturing kernel state at the earliest decision points. This hybrid perspective improves observational fidelity, enabling the reconstruction of multi-step activity chains and providing a more comprehensive view of process and file interactions in cloud-native environments.

### 3.5. Cloud-Native Integration

For monitoring to be effective in cloud-native deployments, kernel-level events must be projected into abstractions that reflect the way workloads are managed and secured, including pods, containers, and namespaces. Without this mapping, raw system calls remain difficult to interpret, especially in multi-tenant clusters where processes from different workloads execute on the same node. Aquila addresses this gap by enriching kernel events at the user-space collector with contextual metadata obtained from the container runtime and the Kubernetes control plane.

**Figure 4:**
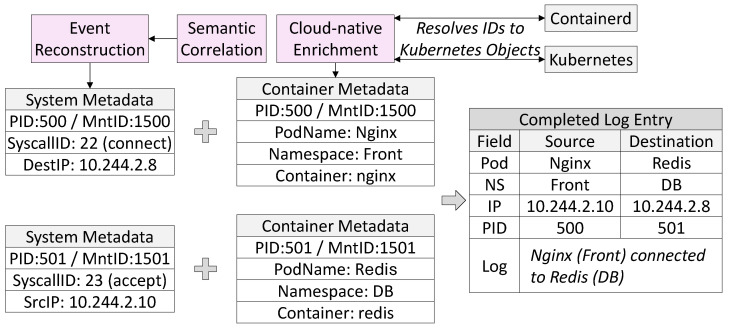
Cloud-native integration in Aquila. Kernel-level metadata is reconstructed and semantically correlated, then enriched with Kubernetes and container runtime context to produce workload-aware logs that map system calls to pods, namespaces, and containers for cluster-wide observability.

As shown in Figure 4, the enrichment process begins with Linux namespace identifiers embedded in kernel event metadata. Each system call record contains the PID and Mount namespace identifiers that link the process to its container. The collector queries the container runtime (e.g., containerd or CRI-O) to resolve this information into a unique container ID. Subsequently, it queries the Kubernetes API server to associate the container with its pod, namespace, and higher-level deployment metadata. In practice, Aquila avoids repeated on-demand queries by continuously watching containerd and Kubernetes events in real time and maintaining the relevant metadata in memory. When a kernel event arrives, enrichment is performed immediately using this cached information, which keeps latency negligible even under high container churn. This multi-stage resolution ensures that every event is expressed in terms that align with Kubernetes abstractions, enabling operators to interpret low-level kernel activity in the same vocabulary used for orchestration and policy.

This design has several benefits. By isolating enrichment in user space, Aquila avoids invasive kernel modifications and depends only on portable eBPF features available in long-term support kernels. This preserves compatibility across heterogeneous infrastructures, ranging from enterprise Linux distributions to managed Kubernetes offerings where kernel upgrades are constrained. The separation also allows enrichment logic to evolve independently of kernel-space probes, enabling rapid adaptation to changes in container runtimes or Kubernetes APIs.

Finally, the enriched events are exported as structured JSON logs or streamed over gRPC, supporting direct ingestion by security information and event management (SIEM) platforms, auditing pipelines, and security policy engines. This standardized interface ensures that kernel-level telemetry is not an opaque trace but a workload-centric signal that integrates seamlessly into existing observability and security ecosystems. In this way, Aquila bridges the gap between low-level system call monitoring and the abstractions that cloud-native operators rely on for managing and securing their clusters.

## 4. Implementation

We implemented Aquila as a combination of eBPF programs and a lightweight user-space collector, validating the design principles outlined in Section 3. The kernel components are written in C (2.1K LoC) using the clang/llvm toolchain, while the user-space collector is implemented in Go (4.4K LoC) via Cilium’s bpf2go, which generates bindings from compiled eBPF object files. This approach preserves portability across kernel versions while supporting long-term support distributions commonly found in production systems.

Kernel-space Implementation. eBPF programs attach to system call tracepoints (e.g., sys_enter_execve, sys_enter_openat) and LSM hooks (e.g., security_bprm_check, security_file_open). Captured events are split into fixed-size metadata and variable-length attributes. Metadata is written to a high-throughput ring buffer, while attributes such as file paths are directed to an auxiliary buffer and linked using 64-bit event identifiers. Critical system calls are routed into reserved-capacity buffers based on a lookup table, whereas non-critical events are stored in best-effort buffers subject to discard. Drop counters maintained in per-CPU maps and embedded sequence numbers allow the user-space collector to detect and quantify loss precisely.

User-space Collector. The collector retrieves events from both channels, reconstructs records by joining metadata with auxiliary attributes, and enriches them with container runtime and Kubernetes metadata. It maintains an in-memory cache of Kubernetes resources updated via watch APIs, avoiding per-event API queries and reducing enrichment latency. This mapping aligns kernel events with pods, containers, and namespaces, making the telemetry actionable in cloud-native settings. Structured outputs are exported as JSON logs, which can be forwarded to security analytics or monitoring backends.

Deployment Model. Aquila is packaged as a Kubernetes DaemonSet to ensure each node runs its own collector and kernel probes. The implementation relies exclusively on portable eBPF features without kernel patches, which enables compatibility across heterogeneous clusters and minimizes operational overhead.

Code Availability. The source code for Aquila is available at https://github.com/boanlab/Aquila (accessed on 19 October 2025). The complete set of experimental scripts used in our evaluation is released in the same repository to support full reproducibility for auditability and reuse.

## 5. Evaluation

This section evaluates Aquila against state-of-the-art eBPF-based monitoring frameworks, including Tetragon, Falco, and Tracee. The evaluation focuses on three primary metrics: processing overhead, event loss rate, and CPU utilization under systematic workloads and realistic system call workloads.

### 5.1. Experimental Setup

All experiments were conducted on a Kubernetes cluster [25] (v1.29.15) consisting of a single node equipped with an Intel Xeon Gold 5220R CPU (Intel Corporation, Santa Clara, United States), 196 GB of memory, and running Linux kernel version 5.15 with eBPF support enabled. We adopted a single-node configuration to isolate per-node monitoring overheads rather than deploying a multi-node cluster, ensuring that the measurements accurately reflect the performance of each framework without cross-node interference. All monitoring frameworks (Tetragon, Falco, Tracee, and Aquila) were deployed as Kubernetes DaemonSets to guarantee consistent deployment and observability settings across experiments.

For a fair comparison, all frameworks were configured to monitor every supported system call rather than relying on default settings, which differ in coverage and granularity. To prevent interference from unrelated workloads, each framework was restricted to observe only the benchmark pod executing the experiments. All logs were written to local files with identical configurations, and the total number of system calls was measured over the entire duration of each benchmark.

System call workloads were generated using the Phoronix OSBench suite [26], which provides microbenchmarks for core kernel activities such as file creation, process and thread creation, program launches, and memory allocation. Each benchmark was executed ten times, and the average per-event processing time was recorded directly from OSBench outputs. The total number of system calls captured by each framework was computed from the corresponding log files under uniform logging configurations.

CPU overheads introduced by each framework were measured at one-second intervals using the Kubernetes metrics server [27]. These measurements were collected while running OSBench workloads at multiple intensity levels, capturing the runtime cost of system call monitoring in terms of resource utilization.

To assess application-level serviceability, we used wrk to generate HTTP workloads with increasing request-per-second (RPS) rates and measured the sustained throughput under each framework. In addition, iperf3 was employed to evaluate the maximum network bandwidth achieved while system call monitoring was enabled, quantifying the impact on end-to-end data-plane performance.

To evaluate performance under realistic conditions, we deployed three representative microservices: AWS’s Retail Store [28], IBM’s Robot Shop [29], and Descartes Research’s TeaShop [30]. Retail Store emphasizes product search, catalog queries, and checkout transactions, making it relatively lightweight and I/O-oriented. Robot Shop combines heterogeneous services in multiple languages with diverse databases, producing bursty requests and complex service interactions. TeaShop emulates an online store and is characterized as a hybrid file and network-intensive workload. These applications reflect execution patterns commonly observed in cloud-native environments.

### 5.2. Structure and Examples of Event Logs

**Figure 5:**
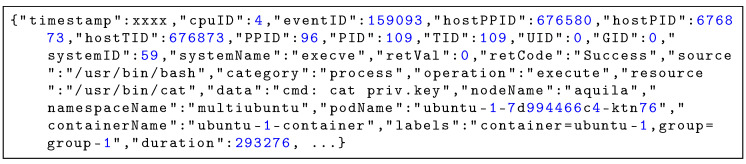
Representative event log entry (execve) captured by Aquila.

**Figure 6:**
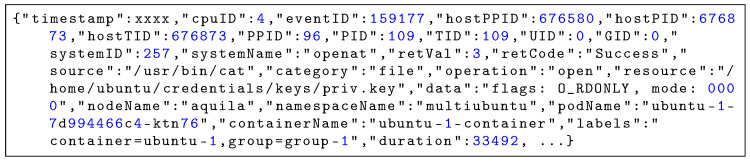
Representative event log entry (openat) captured by Aquila.

To illustrate the raw telemetry collected by Aquila, Figure 5 and Figure 6 show representative event log entries captured during the execution of the cat command accessing a sensitive file. Each event record is serialized in JSON format and consists of several attribute groups. The process context includes identifiers such as PID, TID, PPID, and host-level IDs to reconstruct process ancestry and execution chains. System call information contains the system call name, its arguments, return code, and execution duration, providing low-level visibility into kernel activities. The resource access metadata specifies the operation type (e.g., open, execute), the accessed resource path, and any access flags used. The container and orchestration context adds Kubernetes namespace, pod, container name, and user-defined labels for workload attribution, while node information records the hostname and CPU core ID for correlating events with placement and scheduling decisions. This structured format enables accurate correlation between process activities, system calls, container workloads, and orchestration layers, supporting multi-dimensional security analysis.

### 5.3. Processing Overhead

In this section, we first measure the additional processing overhead introduced by each framework for system calls executed during OSBench workloads.

**Figure 7:**
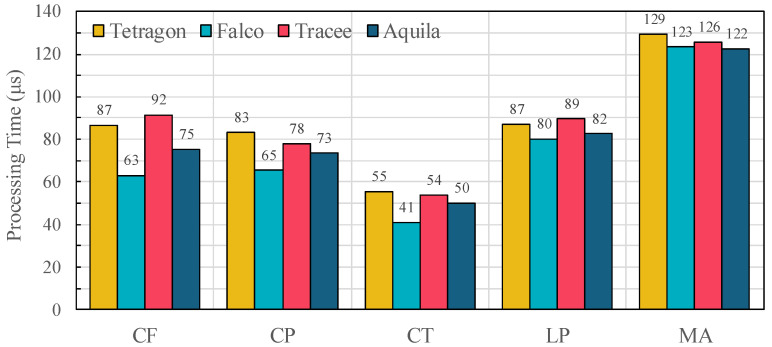
Per-event processing time measured using the Phoronix OSBench suite across different frameworks (Tetragon [12], Falco [11], Tracee [10], and Aquila). Workloads include file creation (CF), process creation (CP), thread creation (CT), program launches (LP), and memory allocation (MA).

Across all workloads in Figure 7, processing latency varies according to the characteristics of each OSBench benchmark. Falco reports the lowest latency for these workloads, averaging 63.1 μs in CF and 65.4 μs in CP, while Aquila records 75.3 μs and 73.4 μs, respectively. Thread creation (CT) shows shorter execution paths, with Falco at 41.0 μs, Aquila at 50.1 μs, and Tetragon at 55.5 μs. Program launches (LP) and memory allocation (MA) exhibit intermediate overheads: Aquila achieves 82.5 μs in LP and 122.5 μs in MA, compared with 89.5 μs and 125.6 μs in Tracee, and 87.0 μs and 129.2 μs in Tetragon. These results show that both the computational complexity of underlying kernel operations and the monitoring architecture influence observed per-event latency.

The architectural designs of Falco, Tracee, and Tetragon explain these trends under our experimental configuration. Falco performs minimal in-kernel processing and relies on a user-space rules engine, reducing processing cost but providing limited buffering guarantees under heavy workloads. Tracee enriches events with container and Kubernetes metadata but processes all events through a single-path buffer without priority separation, leading to higher latency when system call rates surge. Tetragon performs in-kernel metadata attachment for improved attribution, yet lacks priority-aware buffering or detailed loss reporting. In contrast, Aquila combines dual-path event handling, priority-aware buffering, and pre-execution hooks to preserve high-priority events and attach rich context while maintaining competitive performance. Compared with Tracee, Aquila reduces per-event latency by 17.8%, 5.7%, 6.8%, 7.8% and 2.5% across CF, CP, CT, LP, and MA workloads, respectively; reductions compared with Tetragon are 13.1%, 11.6%, 9.7%, 5.1% and 5.2%. These results demonstrate that Aquila achieves a balanced trade-off between monitoring overhead and semantic fidelity, unlike existing frameworks that either prioritize raw performance or incur excessive processing costs.

### 5.4. Event Loss Rate

**Figure 8:**
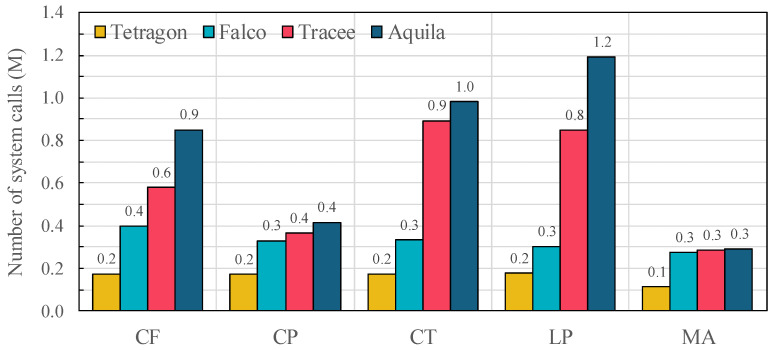
Number of system call events successfully processed under each OSBench workload (CF = Create Files, CP = Create Processes, CT = Create Threads, LP = Launch Programs, MA = Memory Allocation) across evaluated frameworks (Tetragon, Falco, Tracee, and Aquila). (Unit: Million).

Figure 8 reports the number of system call events processed under each OSBench workload. Since the absolute baseline is unknown, Table 5 presents relative loss rates with respect to Aquila, which consistently handles the largest number of events in all workloads. For example, in the LP workload, Tracee, Falco, and Tetragon process 28.8%, 74.5% and 85.1% fewer events than Aquila, respectively. Similar trends appear in CF and CT workloads, where competing frameworks lose between 32–82% more events. Even in the MA workload, where overall event rates are lower, Tracee, Falco, and Tetragon still exhibit 2.3%, 5.8% and 61.0% fewer events than Aquila.

These differences stem from each framework’s buffering and processing design. Falco minimizes in-kernel processing to achieve low per-event latency but lacks backpressure control, causing high losses under bursty conditions. Tracee enriches events with Kubernetes metadata but uses a single-path buffer prone to saturation. Tetragon performs in-kernel metadata attachment yet provides no priority-based buffering or explicit loss detection, resulting in substantial loss under load. In contrast, Aquila integrates dual-path event handling and priority-aware buffering, preserving high-priority events even during overload, and ultimately processes up to 124% more events than competing frameworks.

### 5.5. Resource Utilization

**Figure 9:**
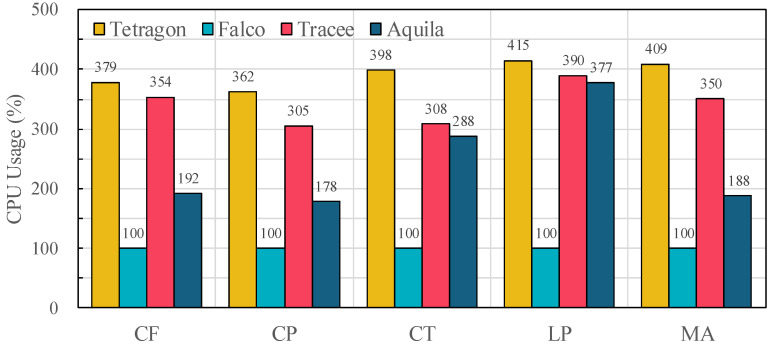
Average CPU utilization under each OSBench workload (CF = Create Files, CP = Create Processes, CT = Create Threads, LP = Launch Programs, MA = Memory Allocation) across evaluated frameworks (Tetragon, Falco, Tracee, and Aquila).

Figure 9 reports CPU utilization incurred by each framework while processing system call events. Falco reports a constant utilization of 100% as its user-space process is single-threaded by default to prioritize stability [31]. This design choice limits concurrency to a single CPU core, which is consistent with the relatively high event loss rates under load [31].

In contrast, Tracee and Tetragon spawn multiple processing threads to handle per-event enrichment and kernel-to-userspace data transfer, which improves concurrency but substantially increases CPU overhead when system call rates surge. For instance, CPU utilization for Tracee and Tetragon exceeds 300% across most workloads, reaching 389.5% and 414.9% in LP, respectively, due to per-event metadata attachment and single-path buffering that amplify contention on the data path. This design explains the moderate-to-high event losses observed in Figure 8 despite higher CPU consumption.

Compared with both frameworks, Aquila consistently lowers CPU usage while sustaining higher event throughput. Reductions versus Tracee are 45.8% (CF), 41.5% (CP), 6.7% (CT), 3.1% (LP), and 46.4% (MA). Reductions versus Tetragon are 49.3% (CF), 50.8% (CP), 27.9% (CT), 9.0% (LP), and 54.1% (MA). These savings stem from Aquila’s dual-path event handling and priority-aware buffering, which minimize unnecessary work on the hot path by separating fixed-size metadata from variable-length attributes and prioritizing critical events under overload. Crucially, Aquila achieves its lower overhead while processing up to 124% more events than competing frameworks (Figure 8), demonstrating a superior balance between resource utilization and telemetry fidelity, unlike Falco’s trade-off of low CPU usage for high event loss.

**Figure 10:**
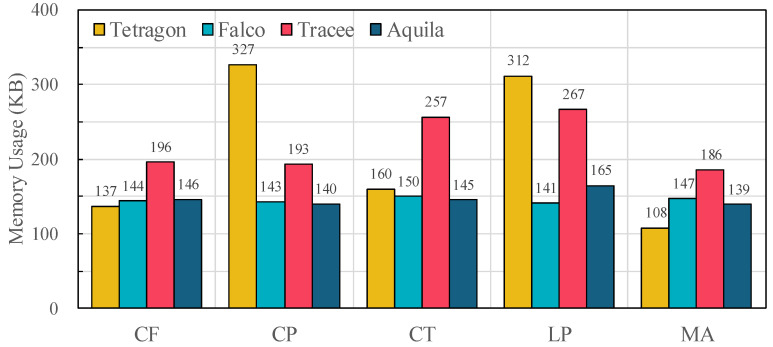
Average memory utilization under each OSBench workload (CF = Create Files, CP = Create Processes, CT = Create Threads, LP = Launch Programs, MA = Memory Allocation) across evaluated frameworks (Tetragon, Falco, Tracee, and Aquila).

Beyond CPU consumption, we also measured memory usage under the same workloads, as shown in Figure 10. Falco maintains a consistently low footprint of around 140–150 KB across all benchmarks, reflecting its lightweight single-threaded pipeline but at the expense of high event loss. Tracee exhibits the largest and most variable memory usage, reaching 257 KB in CT and 267 KB in LP, which indicates additional overhead from concurrent enrichment and buffering. Tetragon also shows significant peaks, especially in CP (327 KB) and LP (312 KB), due to in-kernel metadata attachment without priority separation. By contrast, Aquila maintains a stable footprint between 139 KB and 165 KB across all workloads. This stability arises from its dual-path design, where fixed-size metadata and variable-length attributes are managed in separate channels, preventing buffer saturation and avoiding excessive memory allocation even under intensive workloads.

Taken together with Figure 8, these results demonstrate that Aquila achieves a balanced trade-off: it reduces CPU usage, sustains lower memory overhead than Tracee/Tetragon, and preserves event fidelity under bursty workloads.

### 5.6. Impact on Serviceability

**Figure 11:**
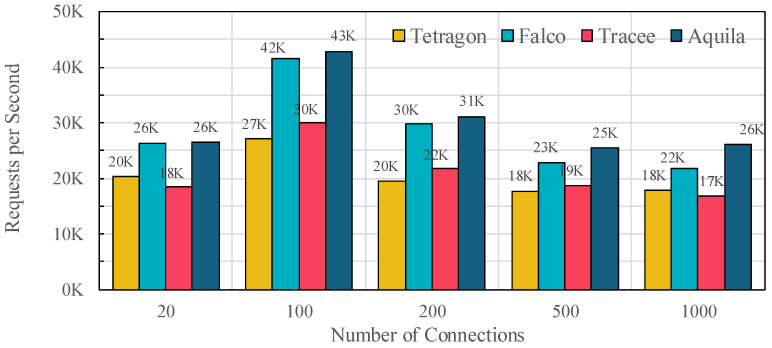
Application-level throughput under increasing offered rates (20–1000 RPS) across Tetragon, Falco, Tracee, and Aquila. Higher values indicate better serviceability.

Figure 11 shows the number of requests processed per second as the offered rate increases from 20 to 1000 RPS. Across all load levels, Aquila sustains the highest service throughput, reaching 26.5 K, 42.9 K, 31.2 K, 25.5 K, and 26.1 K requests at 20, 100, 200, 500, and 1000 RPS, respectively. In contrast, Falco reaches 26.4 K, 41.6 K, 29.9 K, 22.9 K and 21.9 K, while Tracee and Tetragon achieve even lower numbers across all rates.

The throughput gap widens at higher loads. Relative to Falco, Aquila delivers 0.5%, 3.2%, 4.3%, 11.4% and 19.4% more throughput at 20, 100, 200, 500, and 1000 RPS, respectively. Compared with Tracee and Tetragon, the gains range from 36–55% and 30–59%, respectively. These results align with the earlier findings on event loss and CPU overhead: Aquila’s dual-path event handling and priority-aware buffering prevent overload-induced event drops while avoiding unnecessary hot-path work, leading to consistently higher serviceability under realistic production-like conditions.

Finally, Table 6 reports the maximum network throughput measured under constant load. Aquila sustains 21.95 Gbps, slightly higher than Falco (21.70 Gbps) and significantly ahead of Tracee (13.98 Gbps) and Tetragon (12.40 Gbps), confirming that the architectural optimizations in Aquila improve both dynamic and steady-state service performance.

### 5.7. Realistic Deployment Scenarios

To evaluate performance under realistic conditions, we deployed three representative microservices: AWS’s Retail Store, IBM’s Robot Shop, and Descartes Research’s TeaShop. These applications generate heterogeneous system call patterns typical of production environments, including file I/O, process creation, and network communication.

**Figure 12:**
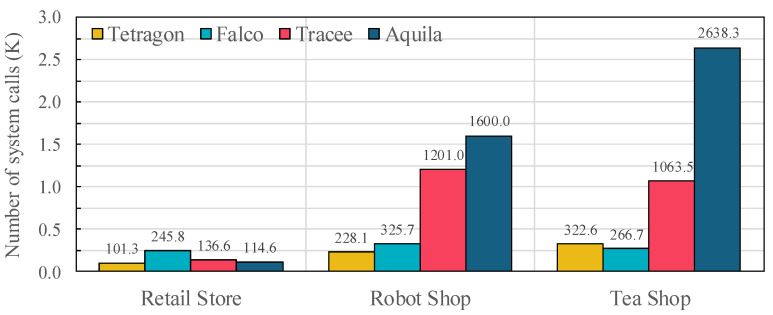
Number of system call events processed under microservice workloads (Retail Store, Robot Shop, TeaShop) across evaluated frameworks (Tetragon, Falco, Tracee, and Aquila).

Figure 12 shows that Aquila sustains the highest event processing rates across all scenarios, capturing 114.6 K, 1600.0 K and 2638.3 K events for Retail Store, Robot Shop, and TeaShop, respectively. In comparison, Tracee records 136.6 K, 1201.0 K, and 1063.5 K events; Falco processes 245.8 K, 325.7 K, and 266.7 K; and Tetragon handles 101.3 K, 228.1 K, and 322.6 K. Two observations emerge. First, the heterogeneous and bursty patterns of Robot Shop and TeaShop expose scalability gaps: compared with Aquila, competing frameworks process 25–88% fewer events, consistent with the relative losses summarized in Table 5. Second, even for the lighter Retail Store workload, Aquila preserves event fidelity while keeping resource usage competitive with the results in Figure 9. These outcomes align with Aquila’s design choices, such as dual-path event handling and priority-aware buffering, which enable millions of events to be captured per workload without sacrificing semantic fidelity or overwhelming CPU resources.

### 5.8. Feature Coverage Comparison

While the previous sections focused primarily on performance and serviceability metrics, we also compare the functional coverage of Aquila with representative eBPF-based telemetry frameworks (Falco, Tracee, and Tetragon). Table 7 summarizes the feature set across key dimensions such as system call coverage, LSM hook usage, container runtime compatibility, and Kubernetes enrichment.

As shown in Table 7, Falco provides broad rule-based coverage with basic Kubernetes metadata support, but lacks explicit loss awareness and deeper enrichment. Tracee achieves extensive system call visibility and offers partial workflow correlation, yet still relies on post-processing for higher-level analysis. Tetragon integrates tightly with Cilium and leverages LSM hooks, but its enrichment is limited to container-level attribution without broader workload context. In contrast, Aquila combines kernel-level fidelity with explicit drop detection, dual-path buffering, and Kubernetes-aware enrichment, enabling both reliable performance and richer semantic observability. This comparison highlights that Aquila’s contributions extend beyond performance efficiency to functional robustness.

## 6. Related Work

System call monitoring has been extensively explored in both systems and security research, evolving from early user-space interception to modern eBPF-based observability and runtime enforcement frameworks. Existing work can be broadly categorized into legacy monitoring approaches, eBPF-based observability tools, and security-oriented enforcement frameworks. Despite significant progress, current solutions continue to face challenges in scalability, reliability, and semantic expressiveness, motivating the design of Aquila.

### 6.1. Legacy System Call Monitoring Approaches

Early mechanisms such as strace and ptrace-based monitors [3,32] offered fine-grained visibility into system call activity but suffered from prohibitive overhead because each invocation triggered additional user–kernel context switches [18]. Kernel-integrated logging facilities, such as auditd [4], reduced interception costs by embedding monitoring hooks inside the kernel itself. However, their coarse-grained filtering frequently forced administrators to trade completeness for performance, resulting in either overwhelming log volumes or insufficient visibility [19].

Instrumentation frameworks such as SystemTap [5] and LTTng [6] introduced dynamic probes to reduce runtime costs, but their dependence on custom kernel modules and debug symbols limited portability and long-term stability in production environments. Similarly, commercial and open-source tools such as sysdig [33] exposed system call events through loadable kernel modules, introducing attack surface expansion and maintenance overhead.

While these approaches established the foundation for system call monitoring, their limited scalability, operational complexity, and inability to handle modern multi-tenant, high-frequency workloads motivated the transition toward safer, more programmable mechanisms such as eBPF.

### 6.2. eBPF-Based Observability Tools

The emergence of eBPF fundamentally changed the monitoring landscape by enabling safe, verifiable, and programmable execution paths inside the Linux kernel. BCC [34] introduced reusable eBPF components for performance profiling, while bpftrace [9] offered a high-level tracing language for ad hoc debugging. These tools significantly lowered the barrier to kernel instrumentation by supporting dynamic attachment, in-kernel filtering, and portability across kernel versions.

However, both BCC and bpftrace were primarily designed for diagnostics and lacked mechanisms for sustained, scalable, low-latency, high-volume telemetry pipelines. Their single-path buffering architectures were prone to overload-induced event loss and provided limited semantic correlation across complex, real-time, multi-step behaviors such as process creation chains or container namespace transitions.

More recent frameworks, such as DeepFlow [35], extended eBPF monitoring to cluster-scale deployments, demonstrating the scalability potential of kernel-resident telemetry. Nevertheless, they retained critical limitations: bounded buffers that silently discard events under bursty workloads, enrichment logic that operates post hoc rather than inline with capture, and minimal correlation across processes, containers, and orchestration layers.

Overall, while eBPF-based observability tools improved performance and portability over legacy solutions, they still lack explicit loss awareness, priority-sensitive buffering, and early semantic visibility. These capabilities are introduced by Aquila through dual-path event pipelines, pre-execution hooks, and reliability-aware buffering.

Beyond these eBPF-based frameworks, recent research has expanded telemetry analysis toward distributed and intelligent operations. Loaiza et al. [36] introduced MLSysOps, demonstrating the use of autonomous agents across the computing continuum for self-adaptive telemetry-driven management. Similarly, Farooq et al. [37] leveraged AI-based forward and backward chaining on telemetry data to enhance diagnostics and prognostics, highlighting the increasing integration of AI reasoning in telemetry pipelines. These studies complement Aquila’s design goal of delivering scalable and context-rich system telemetry for dynamic cloud-native environments.

### 6.3. Runtime Security and Enforcement Frameworks

Several frameworks extend eBPF-based observability toward runtime security and policy enforcement. Tracee [10] enriches system call streams with container and Kubernetes metadata to support threat detection and forensic reconstruction. Falco [11] integrates a rule-based engine for continuous policy evaluation, enabling detection of anomalous behaviors in real time. Tetragon [12] performs in-kernel context enrichment and namespace attribution, reducing latency and improving mapping accuracy between low-level system calls and high-level workload entities.

Complementary approaches such as SELinux [38] and AppArmor [39] enforce mandatory access control policies at the kernel boundary, while projects such as KubeArmor [40] and gVisor [41] explore container-native enforcement through policy translation or sandboxed execution. Despite these advances, most frameworks prioritize detection or enforcement functionality over scalable, loss-aware, and semantically rich telemetry collection.

Aquila bridges this gap by delivering high-fidelity, context-aware system call streams with explicit reliability guarantees, enabling existing policy engines and security analytics frameworks to operate on complete and semantically enriched runtime data without prohibitive performance penalties.

Beyond cloud-native observability, related research in secure distributed systems explores similar challenges. Examples include cross-chain telemetry for blockchain-based metaverse services [42], distributed key generation methods for secure data sharing [43], and explainable time series analysis for anomaly detection [44]. While these domains differ, they share the goal of maintaining reliable, context-rich telemetry under adversarial or large-scale conditions, which aligns with Aquila’s design philosophy.

## 7. Conclusions

This paper introduced Aquila, a system call telemetry framework designed to overcome the systemic limitations of current eBPF-based monitors in cloud-native settings. By combining a dual-path kernel pipeline, priority-aware buffering, explicit drop detection, and user-space enrichment, Aquila achieves a balance between efficiency, reliability, resilience, and semantic expressiveness. Our evaluation demonstrates that the system preserves critical audit trails under stress, reduces per-event overhead through batching and separation of attributes, and provides enriched telemetry that maps kernel-level activity to Kubernetes abstractions. Compared with Tracee and Tetragon, Aquila lowers per-event latency by up to 17.8% and 11.6%, respectively, reduces CPU usage by more than 50% under OSBench workloads, and sustains up to 124% more events under load, confirming both efficiency and scalability advantages. These contributions make Aquila a practical and deployable solution for large-scale clusters that require both high-fidelity security monitoring, operational scalability, and adaptive workload management.

Future work will focus on extending the enrichment pipeline to support distributed correlation across multi-node clusters, validating scalability under cross-node workload migration, and integrating adaptive buffering policies. We also plan to investigate the resilience of the priority mechanism against adversarial manipulation and to explore defenses such as system call rate limiting and anomaly-aware reprioritization. An in-depth security analysis of priority-aware buffering under adversarial workloads is left for future work. We plan to evaluate mitigation strategies such as dynamic reprioritization, rate-limiting of anomalous processes, and integration with kernel-level anomaly detectors to strengthen resilience against priority inversion attacks.

## Figures and Tables

**Table 1:** Comparison of system call monitoring approaches.

Approach	Mechanism	Strengths	Limitations
ptrace/strace	User–kernel interception	Simple to deploy, fine-grained visibility	Severe overhead due to extra context switches
auditd	Kernel-integrated logging	Standardized, secure logging	Coarse-grained filtering, high log volume
SystemTap/LTTng	Kernel probes and tracepoints	Lower overhead, detailed tracing	Requires modules/debug symbols, complexity
sysdig	Loadable kernel module	Configurable filters, detailed tracing	Expands attack surface, portability concerns
eBPF (modern)	Verified in-kernel programmability	Safe, flexible, low-overhead, dynamic	Scalability and semantic context remain open

**Table 2:** Key challenges in current eBPF-based system call monitoring frameworks.

Challenge	Root Cause	Implications in Cloud-Native Environments
Performance overhead	Kernel-to-user event construction, serialization, and enrichment	CPU saturation; latency overhead in CI/CD pipelines and I/O-intensive workloads
Event loss/backpressure	Bounded perf/ring buffers; lack of prioritization	Missing audit trails; forensic blind spots; adversarial flooding attacks
Limited semantic context	Per-event perspective; no long-lived in-kernel correlation	Difficulty reconstructing multi-step attack chains; reactive detection pipelines

**Table 3:** Comparison of system call monitoring and security frameworks.

Framework	Focus	Performance Overhead	Event Loss Handling	Semantic Context	Cloud-Native Integration
strace/ptrace	Legacy tracing tools	High	None	Low	No
auditd	Kernel-integrated logging	Medium	None	Low	No
SystemTap/LTTng	Debugging/tracing	Medium	Limited	Low	No
Tracee/Falco	Security monitoring	Medium	None	Medium	Partial
Tetragon	Observability + in-kernel enrichment	Medium	Limited	Medium	Yes
Aquila	Performance + fidelity + reliability	Low	Explicit + Priority	High	Yes

**Table 4:** Examples of security-critical system calls.

Category	System Call(s)	Main Signals
Execution	execve, execveat	New binary execution, privilege transitions
Process	clone, fork, vfork, clone3	Process tree branching, namespace isolation
File Open	open, openat	Critical file creation/destruction, configuration changes
File Permission	chmod, fchmod, chown, fchown	Permission escalation
File Modification	rename, unlink, unlinkat	File stealth, evidence deletion
Privilege	setuid, setgid, setresuid, setresgid	Privilege escalation, identity switching
Memory	mmap, mprotect	RWX memory region creation, JIT attacks
Network	socket, connect, accept	Outbound connections, C&C traffic
Binding	bind, listen	Unauthorized service exposure
Namespace	setns, unshare	Namespace transitions, container escape attempts
Capabilities	capset	Capability escalation
Security Policy	prctl, seccomp	Policy bypass, seccomp weakening
Injection/Tracing	ptrace	Process injection, debugging attachment

**Table 5:** Relative event loss rates compared with Aquila across OSBench workloads. Lower is better.

Workload	Tracee	Falco	Tetragon
CF	32.0%	53.2%	79.4%
CP	12.2%	20.6%	58.6%
CT	8.9%	66.1%	82.2%
LP	28.8%	74.5%	85.1%
MA	2.3%	5.8%	61.0%

**Table 6:** Network throughput (Gbps) under constant load.

	Tetragon	Falco	Tracee	Aquila
Throughput (Gbps)	12.40	21.70	13.98	21.95

**Table 7:** Feature coverage comparison of system call telemetry frameworks.

Feature	Falco	Tracee	Tetragon	Aquila
System call Coverage	Broad, kernel tracepoints	Extensive, raw system calls	Selective, focused onsecurity-relevant system calls	Extensive, withpriority-aware filtering
LSM Hook Support	No	Limited (experimental)	Yes	Yes
Container Runtime	Docker, containerd, CRI-O	Docker, containerd	containerd (Cilium integration)	Docker, containerd
Kubernetes Integration	Pod/Namespace	Container Only	Full enrichment	Full enrichment
Event Loss Awareness	None	None	Partial (BPF counters)	Explicit drop detectionwith per-CPU counters
Buffering Strategy	Single, perf buffer	Single, perf buffer	Ring buffer withperf fallback	Dual-path, priority-aware
Semantic Correlation	Rule-based filtering	system call-level stream	Limited (per-event analysis)	Multi-step workflow reconstruction

## Data Availability

The complete set of experimental scripts used for the evaluation is available at https://github.com/boanlab/Aquila (accessed on 19 October 2025).

## Abbreviations

The following abbreviations are used in this manuscript:

eBPF	Extended Berkeley Packet Filter
LSM	Linux Security Module
CI/CD	Continuous Integration/Continuous Deployment
C&C	Command and Control
PID	Process Identifier
TID	Thread Identifier
JSON	JavaScript Object Notation
